# Mineral Mimetic Material Sr-Exchanged Sitinakite of Different Crystallinity: Phase Transformations during Heat Treatment and the Strength of SR Fixation in a Ceramic Matrix

**DOI:** 10.3390/ma17091991

**Published:** 2024-04-25

**Authors:** Igor A. Perovskiy, Dmitry A. Shushkov, Alexey V. Ponaryadov, Galina O. Kalashnikova, Ayya V. Bazai, Vladimir N. Bocharov, Taras L. Panikorovskii

**Affiliations:** 1 Institute of Geology of Komi Science Centre of Ural Branch of the Russian Academy of Sciences, 167000 Syktyvkar, Russia; dashushkov@geo.komisc.ru (D.A.S.); alex401@rambler.ru (A.V.P.); 2 Laboratory of Nature-Inspired Technologies and Environmental Safety of the Arctic, Nanomaterial Research Center of the Kola Science Centre, Russian Academy of Sciences, Fersmana Str. 14, 184209 Apatity, Russia; t.panikorovskii@ksc.ru; 3 Laboratory for Synthesis and Research of the Properties of Mineral-like Functional Materials, Nanomaterial Research Center of the Kola Science Centre, Russian Academy of Sciences, Fersmana Str. 14, 184209 Apatity, Russia; g.kalashnikova@ksc.ru (G.O.K.);; 4 Saint-Petersburg State University, Universitetskaya Emb., 7/9, 199034 St. Petersburg, Russia; regbvn@gmail.com

**Keywords:** titanosilicate, sitinakite, sorption, strontium immobilization, ceramics, Synroc

## Abstract

A simple method for the direct transformation of Sr-exchanged titanosilicate with the sitinakite structure (IONSIV) into ceramic material through cold pressing and subsequent sintering at 1100 °C for 4 h is presented. The temperature transformation of Sr-exchanged sitinakite showed the stages of recrystallization of the material with the formation of Sr-Ti phases matsubaraite (Sr_4_Ti_5_[Si_2_O_7_]_2_O_8_), jeppeite (SrTi_6_O_13_), tausonite (SrTiO_3_), and rutile. Leaching experiments showed the efficiency of fixation of Sr cations in a ceramic matrix; extraction into water does not exceed 0.01% and desorption in 1 M HNO_3_ solution is only 0.19% within three days. The leaching rates of immobilized Sr demonstrate the structural integrity of the formed phases in the ceramic (2.8 × 10^−5^–1.0 × 10^−5^ g/(m^2^·day). The decrease in the crystallinity of the initial Na-sitinakite, which is achieved by reducing the synthesis temperature from 250 to 210 °C, does not affect the sorption capacity and the fixation of cations in the ceramic matrix. The obtained results confirm the prospect of using inexpensive precursors, titanium ore enrichment waste, for the synthesis of sorption materials.

## 1. Introduction

Mineral mimetic materials have many different applications in modern industry. One of the urgent problems in the use of radioactive substances in various industries, most importantly in the nuclear industry, is the accumulation of a significant amount of radioactive waste. Liquid radioactive waste (LRW) of low and medium activity level is an environmental hazard due to its large volume, high total activity, and possibility of uncontrolled dispersal in the case of emergencies.

The radionuclides ^90^Sr and ^137^Cs make a significant contribution to total LRW activity. The toxicity of ^90^Sr is associated with the easy substitution of calcium for strontium in bone tissue and the development of strontium rickets, as well as with their subsequent internal exposure leading to leukemia and sarcoma [1]. Due to its good solubility, ^137^Cs is able to move freely in aqueous media, and when ingested into the human body, it is easily reabsorbed in the intestine and evenly distributed throughout the tissues. As a result of the uniform distribution of nuclides in tissues, all organs suffer from irradiation. High doses of ^137^Cs can cause medullary dystrophy, reproductive disorders, and unfavorable effects on liver and kidney function [2,3].

At the moment, the most promising solutions for handling such waste are decontamination technologies using sorption materials of various types: organic ion exchange resins, aluminosilicate sorbents, and inorganic sorbents based on ferrocyanides of transition metals [4,5,6,7]. Recently, mineral mimetic materials have been included among such applications [8,9,10,11]. The most used for the selective extraction of radionuclides ^137^Cs and ^90^Sr from LRW is the molecular sieve IONSIV-911, produced by the American corporation UOP Molecular Sieves [12,13,14,15,16]. This compound is a synthetic analog of natural sitinakite discovered in the Khibiny massif [17]. IONSIV-911 is an ion exchanger material that has been shown to be highly effective for processing LRW in a variety of environments (Fukushima Daiichi, the Three-Mile Island, and the Savannah River Plant) [18,19].

We have developed a technology for the production of sitinakite (IONSIV) using an inexpensive precursor—the enrichment waste of quartz–leucoxene concentrates [20]. The use of enrichment waste instead of expensive traditional precursors (titanium isopropyloxide Ti(OC_3_H_7_)_4_, TiOCl_2_, TiCl_4_, and TiCl_3_), which make the main contribution to the cost of sorbents, allows to significantly reduce the cost of titanosilicate production. The titanosilicate synthesized on the basis of enrichment waste showed high efficiency in extraction cations with charges of 1–2 and 3 (Cs, Sr, Ba, La) from model aqueous solutions [21,22].

The interest in titanosilicates is conditioned not only by their high sorption characteristics but also by the possibility of obtaining from them Synroc ceramics, which is a durable, resistant to radioactive self-exposure, and acid-leaching material. The basis for the production of Synroc ceramics, developed at the Australian National University [23,24,25,26,27,28,29], is the hot isostatic pressing of a mixture of titanium, calcium, zircon, barium, and aluminum oxides. The reaction product is ceramics consisting of synthetic analogs of the minerals perovskite, zirconolite, hollandite, and rutile. The obtained ceramics show good results regarding the fixation of uranium decay products in ceramic matrices, and the modification of ceramics through the introduction of additional components contributes to the improvement of durability characteristics and a reduction in radionuclide leaching [30,31,32,33,34]. The difficulty of obtaining Synroc ceramics is the need for homogeneity in the starting mixture of oxides since the inhomogeneity of the material can lead to incomplete incorporation of the radionuclide into the ceramic matrix [34]. The prospects of the fixation of some cations (Sr, Cs, Am, Eu, and Cm) in the matrix obtained from titanosilicate sorbents have been considered in some articles [35,36,37,38,39]. The results presented in the articles show that the use of titanosilicates for the selective sorption of radionuclides from LRW will not only reduce the amount of waste but also simplify the conditions for obtaining Synroc titanate ceramics.

This article presents the results of obtaining titanate ceramics of Synroc type, which are formed through the sintering of Sr-exchanged sitinakite. Sr-exchanged sitinakite was prepared by cation exchange from Na-sitinakite, which was synthesized using titanium ore enrichment waste. The strength of Sr fixation in the ceramic matrix following water and acid treatment was evaluated and the influence of the crystallinity of the initial Na-sitinakite on the phase composition of the final ceramic was shown.

## 2. Materials and Methods

### 2.1. Synthesis of Na-Sitinakite

Na-sitinakite (an analog of natural sitinakite without K) synthesis was performed using, as a precursor, hydrated sludge obtained from recycled solutions of fluorammonium technology of quartz-leucoxene (titanium) concentrate processing [40] The obtainment of hydrated sludge containing wt (%) 49.0—TiO_2_; 45.8—SiO_2_; 4.6—Fe_2_O_3_; 0.3—Al_2_O_3_; 0.1—CaO; and K_2_O is described elsewhere [41]. The dried hydrated precipitate of 0.5 g was treated with 37 mL of 1 M NaOH solution and dispersed for 20 min with a magnetic stirrer. The final mole ratio of TiO_2_:SiO_2_:Na_2_O:H_2_O in the resulting alkali titanium–silicon mixture was 1:1.22:6:671.2. The obtained mixture was transferred into a Teflon-lined autoclave (45 mL, filling degree 80%) and heated for 12 h. After cooling down to room temperature naturally, the product was centrifuged and washed with distilled water (450 mL) until pH 5.6–6. The obtained sample was dried at 103 °C for 4 h. To reveal the dependence of the titanosilicate degree of crystallinity on sorption, synthesis was carried out at temperatures of 210 and 250 °C. The samples were designated as T-210-Na and T-250-Na, respectively).

### 2.2. Synthesis of Sr-Exchanged Forms of Sitinakite

To obtain Sr-exchanged sitinakite, a series of experiments on ion exchange on starting sitinakite with different crystallinity (T-210-Na and T-250-Na) were carried out. Ion exchange was carried out in a model solution with a Sr concentration of 2 g/L prepared by solubilizing strontium nitrate (Sr(NO_3_)_2_, Merck, ≥99.0%) in deionized water (10 MΩ∙cm). The starting pH of the solutions was 5.1. The ratio of solid sorbent to liquid phase was 1:250, the sorption temperature in static mode was 20 °C, and the sorption time was 24 h with periodic shaking. At the end of the process, the suspension was centrifuged at 3000 rpm for 5 min, and after sorption, an aliquot was taken to characterize the sorption properties of the synthesized material.

According to the obtained data, the sorption capacity (Q, mg/g) of the obtained sorbents was calculated according to the equation:

$$Q=\frac{\left(C_{o}-C_{e}\right)V}{m}$$

where C_O_ and C_e_ correspond to the initial and equilibrium concentrations of ions in solution, mg/L; V is the volume of solution, L; and m is the sorbent sample weight.

The separated precipitate was washed with 100 mL of deionized water and dried at 105 °C for 4 h. The temperature effects of Sr-exchanged sitinakite were studied using the DTA method. To obtain information on phase transformations during temperature heating, Sr-exchanged sitinakite was successively heat-treated at temperatures of 300, 650, 850, and 1100 °C at a heating rate of 10 °/min and held for 1 h at a given temperature. The heat-treated material was analyzed using powder X-ray diffraction on a low-background silicon holder.

### 2.3. Mechanism of Sr^2+^ Incorporation into the Structure of Sitinakite

The mechanism of Sr^2+^ incorporation into the sitinakite structure was investigated in an ion exchange experiment using natural sitinakite, followed by a crystal structure study on a single crystal diffractometer (Bruker Apex II, Karlsruhe, Germany). A sample of sitinakite with a chemical composition corresponding to the formula (Na_2.28_K_0.68_Sr_0.06_Ba_0.02_Ca_0.01_)_3.05_(Ti_3.82_Nb_0.25_Fe_0.01_)_4.08_Si_1.94_O_13_(OH_0.54_O_0.46_)·3.98H_2_O was used in the exchange experiment. The exchanged forms of natural sitinakite for the monocrystal study were obtained at 180 °C for 24 h. After the experiment, the sample was kept at room conditions for 90 days. The chemical composition of the Sr-exchanged sample was (Sr_1.61_Na_0.20_)_1.81_(Ti_3.61_Nb_0.38_Fe_0.10_)_4.09_Si_2.01_O_13_(O)·5.25H_2_O.

### 2.4. Titanate Ceramic Synthesis and Leaching Test

To evaluate the strength of strontium fixation in the ceramic matrix (leaching test) the samples of titanate ceramics were prepared based on Sr-exchanged sitinakite (samples T-210-Sr and T-250-Sr). Tablets were pressed using a manual hydraulic press at a pressure of 2 MPa. The obtained tablets were calcined in air atmosphere at 1100 °C. The heating rate was 10 °C/min and the isothermal holding time was 4 h. The efficiency of Sr fixation in ceramics was evaluated by its content in extracts obtained through two-stage treatment: (1) the material was kept in deionized water (pH = 5.1 ± 0.1) and then (2) the ceramics were placed into 1 M nitric acid solution. In all experiments, the amount of extractant was 0.01 L, the extraction time at each stage was 3 days with aliquot sampling on day 1 and day 3, and desorption was at room temperature 25 ± 2 °C.

The normalized leaching rate was determined according to the equation:

$$NRi=\frac{C_{i}\cdot V}{(Q\cdot m)\;\cdot SA\cdot t}$$

where NRi—normalized leaching rate (g/(m^2^·day)), C_i_—element concentration (g/L) in solution after leaching, V—volume of solution for leaching (L), Q—sorption capacity (mg/g), m—mass of the ceramic sample before calcination (g), SA—area of the ceramic sample (m^2^), and t—leaching duration (in days).

### 2.5. Material Characterizations

The chemical composition of hydrated powders (precipitate) was determined using X-ray fluorescence analysis on the CleverA17 instrument JSC “ELERAN” (Moscow, Russia). Phase analysis of the synthesized Na-sitinakite, its Sr-exchanged form, as well as all temperature transformations was carried out using X-ray diffraction with the diffractometer XRD-6000 Shimadzu (CuKα radiation in the range of reflection angles 2θ of 2 to 60°). The synthesized phases were determined using the ICCD PDF-4 database. The size of the coherent scattering region (CSR) in the obtained material samples was estimated by the width of diffraction lines at half height using the Selyakov–Sherrer equation. The chemical composition of sitinakite was characterized using a TESCAN VEGA 3 LMH scanning electron microscope with an X-Max energy dispersive detector (Oxford Instruments, Abingdon, UK) at an accelerating voltage of 20 kV.

Adsorption and textural properties were evaluated via low-temperature (−196 °C) nitrogen adsorption–desorption measured using the volume method on a NOVA 1200e (Quantachrome, Boynton Beach, FL, USA) surface area and porosity analyzer. Prior to analysis, the samples were degassed in vacuum at 110 °C for 2 h. The specific surface area was determined using the BET method. The single-point method was used to calculate both adsorption pore volume (V_spads_) and adsorption average pore diameter (D_spads_). The relative error in determining the pore volume was ±1% and that of the surface area and pore size was ±10%. 

The content of elements in the model solution before and after sorption on sitinakite, as well as the content of elements in solutions after leaching from ceramics, was carried out on an Agilent 7700 (Santa Clara, CA, USA) inductively coupled plasma mass spectrometer. Differential thermal analysis was carried out on a TGA/DSC 3+ (Mettler Toledo, Greifensee, Switzerland), with a temperature range of 25 to 1100 °C, a heating rate of 10 °C/min, and an air environment. The morphology and composition of ceramics were studied using a scanning electron microscope, TESCAN VEGA 3 LMH (Brno, Czech Republic). The zeta potential and pH of the isoelectric point were measured with Zetasizer Nano ZS equipment (Malvern Instruments Ltd., Malvern, UK). The measurements were carried out in absence of a background electrolyte in the pH range from 2 to 10 (sample weight 0.04 g; solution volume 25 mL).

For the single-crystal XRD study, several crystals of sitinakite were kept in 1 M Sr(NO_3_)_2_ solution (10 mL) for 24 h at 200 °C without periodic shaking in hermetically sealed autoclaves for hydrothermal synthesis (TOPT-HT10, Toption Instrument, Zhengzhou, Xian, China). After the removal of the Sr(NO_3_)_2_ solution with a Pasteur pipette, the crystals were washed with a three-fold volume of distilled water and dried in air for 2 h. 

The Raman spectra of sitinakite and the Sr-exchanged form collected from uncoated individual grains were recorded with a Horiba Jobin-Yvon Lab RAM HR 800 spectrometer (Longjumeau, France) equipped with an Olympus BX-41 microscope in backscattering geometry (Saint-Peterburg State University, Saint-Peterburg, Russia). The Raman spectra were excited by a solid-state laser (532 nm) with an actual power of 2 mW under the 50× objective (NA 0.75). The spectra were obtained in the range of 70–4000 cm^–1^ at a resolution of 2 cm^−1^ at room temperature. To improve the signal-to-noise ratio, the number of acquisitions was set to 15. The spectra were processed using the algorithms implemented in Labspec and Origin Pro 8.1 software packages.

The single-crystal X-ray diffraction studies were performed at the Resource Center for X-ray Diffraction Studies of St. Petersburg State University on a Bruker Apex II diffractometer. More than a hemisphere of diffraction data was collected using Mo*K*α radiation (scanning step 1°; exposure time 10 s). The unit cell parameters were determined and refined through the use of the least-squares method for 964 reflections. The data were integrated and corrected by means of the CrysAlis Pro program package, which was also used to apply empirical absorption correction using spherical harmonics, implemented in SCALE3 ABSPACK scaling [42]. The structure was solved using SHELXT and refined via the SHELXL software package [43]. The crystal structure was finally refined in the *Cmmm* space group with *R*_1_ = 0.061 for 964 independent reflections with (|*F*o| > 4σ*F*). The crystallographic parameters and structure refinement parameters are given in Table 1, the atomic coordinates and isotropic atomic displacement parameters are given in Table A1, the anisotropic parameters of atomic displacement parameters are given in Table A2, and the selected bond lengths are given in Table A3.

## 3. Results

### 3.1. Titanosilicate Characterization

**Figure 1:**
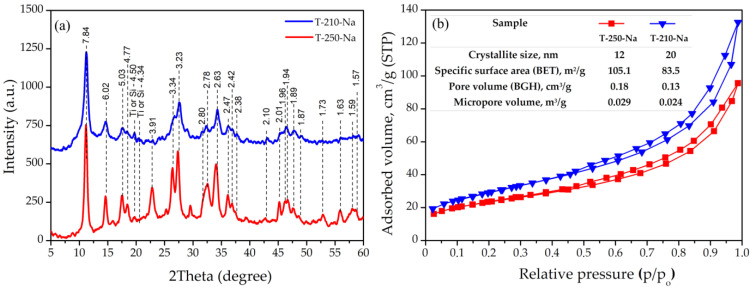
X-ray diffraction (**a**) and N_2_ adsorption–desorption isotherms (**b**) of Na-sitinakite samples synthesized at 210 and 250 °C.

The study of the synthesis product obtained at 250 °C through X-ray phase analysis showed the presence of tetragonal sitinakite with space group P4_2_/mcm (PDF Card No. 00-050-1689), which was identified by a series of reflections with interplanar distances d/n (Å): 7.84, 6.02, 5.03, 4.77, 3.91, 3.34, and 3.23 (Figure 1a). Broadening of the main reflections of sitinakite is characteristic of synthesis products obtained at 210 °C, probably related both to the local disorder of its crystal structure and the formation of nano-sized crystallites. The crystal size decreases from 20 to 12 nm when the synthesis temperature decreases from 250 to 210 °C (Figure 1b). The diffraction patterns of samples T-250-Na and T-210-Na show weak reflections with interplanar distances of 4.54 and 4.50 Å, which can be attributed to both silicon dioxide (PDF Card No. 00-040-1498) and titanium dioxide (PDF Card No. 01-081-9508). The intensity of these reflections in sample T-250-Na is lower than in sample T-210-Na.

The N_2_ adsorption–desorption isotherms for Na-sitinakite and the calculated textural parameters are shown in Figure 1b. With decreasing crystallite size, an increase in both the specific surface area of the material and the total pore volume and micropore volume is observed.

The EDS analysis demonstrates increased content of SiO_2_ in the system in comparison with the theoretical ratio of basic elements for samples T-250-Na and T-210-Na (Table 2). The content of TiO_2_ in the samples is lower than the theoretical value. The admixture of Fe in the synthetic sitinakite can be explained by substitution, which has been observed in natural samples: Fe^3+^ + M^2+^ = Ti^4+^ + M^+^, where M^2+^ = Ca, Ba, and Sr and M^+^ = Na and K [17]. The presence of impurities characterized by the same content of TiO_2_ and SiO_2_ confirms the presence of an extrinsic phase in the synthesis products, which are difficult to identify using X-ray diffraction (Table A4).

**Figure 2:**
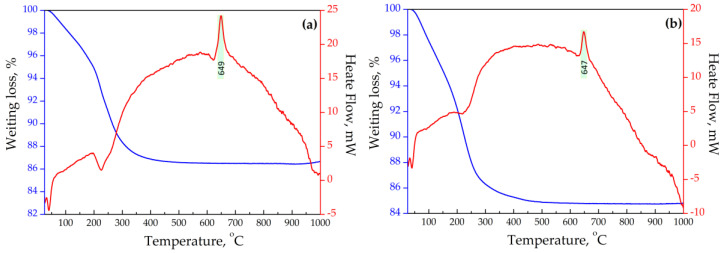
DTA and TG curves of synthesized titanoilicates: (**a**)—sample T-250-Na and (**b**)—sample T-210-Na.

The main mass loss during the heating of initial Na-sitinakite falls in the temperature range of 25 to 300 °C (Figure 2). In this range, physically adsorbed water is removed. At the same time, the T-210-Na sample is characterized by a greater mass loss up to a temperature of 200 °C, which is a consequence of the increased surface area and moisture absorption activity of the sample. Temperature increases up to 300 °C lead to the partial removal of hydrate (structural) water associated with exchangeable cations. 

**Figure 3:**
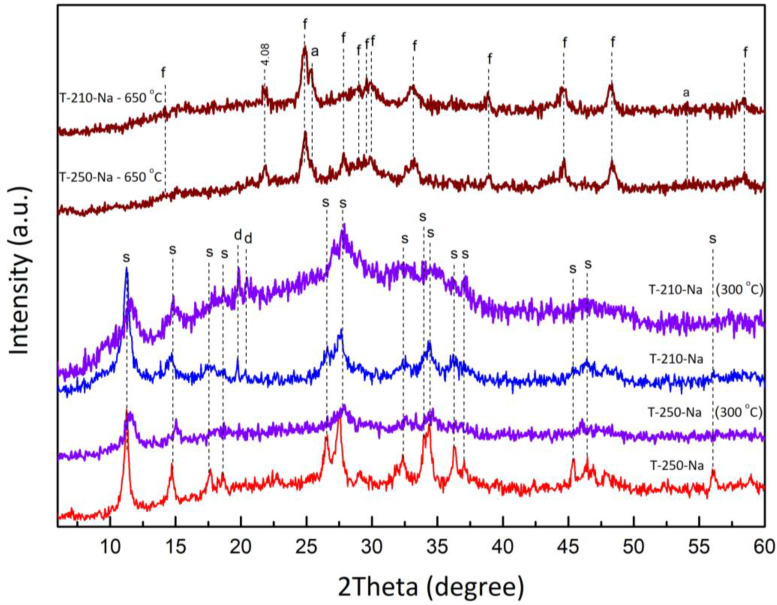
Phase transformations of T-250-Na and T-210-Na sitinakite samples during heat treatment at 300 and 650 °C (s—sitinakite; d—titanium dioxide; f—freidenbergite; a—anatase).

On the diffractograms of samples T-210-Na and T-250-Na, there is a decrease in the intensity of reflections and their broadening; however, complete amorphization does not occur (Figure 3). Resistance to thermal influence is a favorable factor that allows for the effective use of synthesized samples of sitinakite for radionuclide extraction from LRW since the temperature in separate layers of LRW is increased by 130 °C.

**Figure 4:**
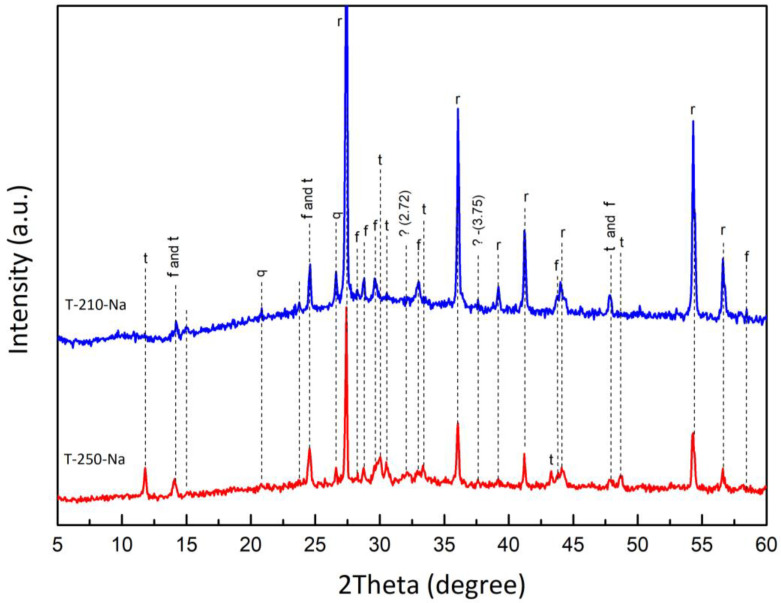
Phase compositions of samples T-250-Na and T-210-Na upon 1000 °C sintering (r—rutile; f—freidenbergite; t—sodium titanate; q—quartz).

Further heat treatment leads to the loss of chemically bound water included in the crystal lattice of sitinakite (including the hydroxyl group OH^–^) with amorphization of titanosilicate and strong fixation of exchangeable Na^+^ cations inside the framework. The exothermic peak at 650 °C corresponds to the crystallization of freidenbergite (Na_2_Ti_6_(Fe^3+^,Si)_2_O_16_). Sintering at a temperature of 1000 °C promotes the increase in the amount and crystallinity of freidenbergite, as well as the formation of quartz and anhydrous titanate (Na_2_Ti_6_O_13_, PDF Card No 00-037-0951), the reflections of which were identified in sample T-250-Na (Figure 4).

**Figure 5:**
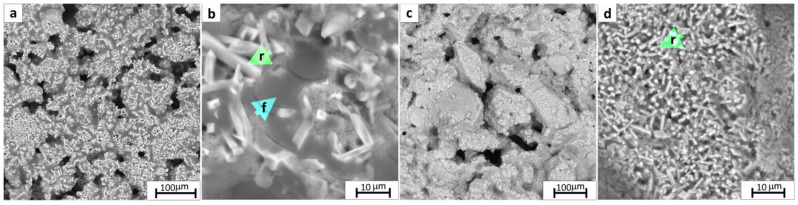
SEM images of the sintered material obtained at 1000 °C of T-250-Na (**a**,**b**) and T-210-Na (**c**,**d**) during thermal analysis, BSE mode (r—rutile; f—freidenbergite).

The sintering of powders at a temperature of 1000 °C leads to the formation of columnar crystals of rutile immersed in the melt, whose composition is close to freidenbergite (Figure 5). Sample T-210-Na, calcined at 1000 °C, is characterized by smaller sizes of rutile crystals, which is most likely connected to the dimensional characteristics of the initial powdered material. Also, it is possible to note the increase in the intensity of rutile reflections in the diffraction pattern of the T-210-Na sample, which can be connected to the formation of the ordered crystal lattice in smaller crystals (Figure 4).

### 3.2. Ion-Exchange-Induced Transformation

The results of Sr^2+^ sorption on sitinakite are presented in Table 2. The sorption capacity of sample T-250-Na was 107 mg/g. The decrease in crystallinity in sample T-210-Na did not affect the sorption capacity, which reached 112 mg/g. The results for the area elemental composition from the EDS data of sitinakite samples before and after sorption are presented in Table 2. It should be noted that not all Na participates in the sorption process.

**Figure 6:**
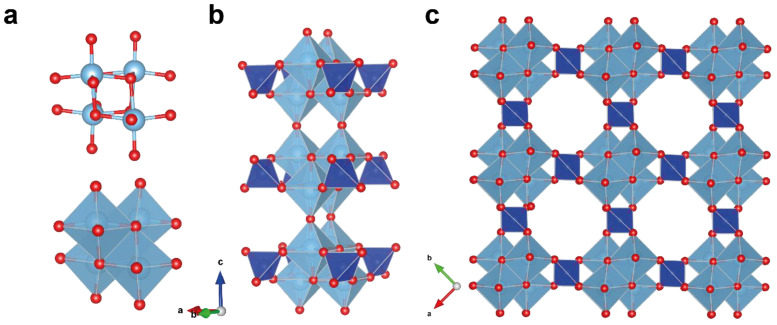
Projection of the crystal structure framework of sitinakite along the c-axis (**a**); [Ti_4_O_4_]^8^_+∞_ columns with adjacent SiO_4_ tetrahedra (**b**); [Ti_4_O_4_]^8+^ cubane-like clusters in sitinakite (**c**).

The sitinakite structure is based on [Ti_4_O_16_] tetramers formed by edge-shared Ti-centered octahedrons (Figure 6a). The tetramers are connected to each other through common vertices along [001] and by common vertices with SiO_4_ tetrahedrons along the directions [100] and [010] into a framework (Figure 6b). The heteropolyhedral sitinakite framework contains a three-dimensional system of crossed channels oriented along the main crystallographic directions of the tetragonal cell, with a maximum free crystallographic radius of ~3.5 Å (Figure 6c); these channels are filled with Na^+^, K^+^ ions, and H_2_O molecules and can be replaced by large Cs^+^ and Sr^2+^ [44].

**Figure 7:**
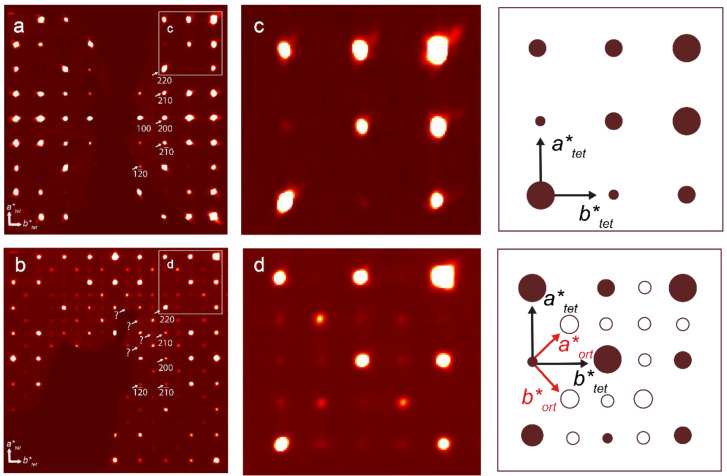
Reconstructed reciprocal cross-sections obtained for the (*hk*0) section of sitinakite (**a**) and its Sr-exchanged form (**b**) and enlarged fragments of these cross-sections (**c**,**d**). White arrows and numbers indicate reflections and their indices. Examples of additional reflections that cannot be indexed in the tetragonal cell are indicated by question marks. In the corresponding schematics, large dark red circles and small blank circles refer to the tetragonal and orthorhombic cells, respectively; black and red arrows indicate the vectors of the tetragonal and orthorhombic cells, respectively.

An attempt to refine the crystal structure of Sr-exchanged sitinakite in the same space group as the original sitinakite (*P*4_2_/*mcm*) was unsuccessful; the structure was refined in the *P*−4_2_*m* space group with a value of *R*_1_ = 0.113 for 2673 (*R*_int_ = 0.073, *R*_sigma_ = 0.039) independent reflections. In this space group, we observed a large number of atoms with physically unrealistic displacement parameters and the presence of 1476 unindicated reflections (*I* > 3σ(*I*)). Careful inspection of the additional reflections revealed the *Cmmm* space group as the most probable for Sr-exchange sitinakite. A new unit cell setting was used to account for the additional reflections (Figure 7). The crystal structure was refined using a merohedral twinning model (the second order axis along [110]) with a twin ratio of 0.451/0.549 with *R*_1_ = 0.059 for 964 unique observed reflections with |*Fo*| ≥ 4σ.

**Figure 8:**
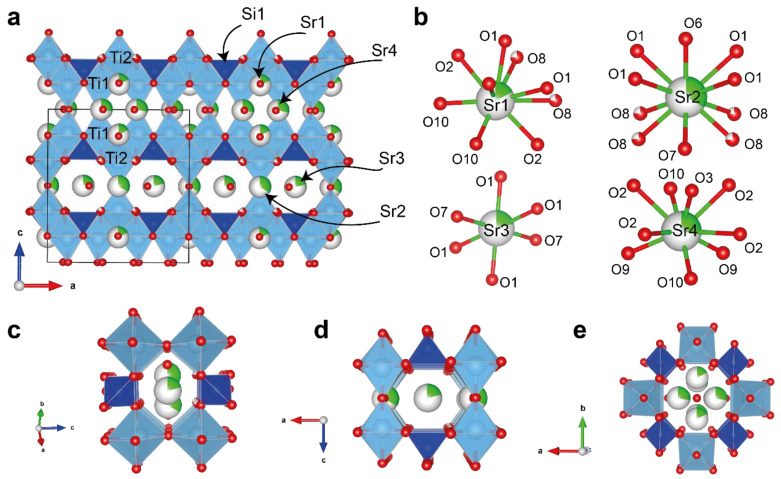
Crystal structure projection of Sr-substituted sitinakite along the b-axis (**a**); coordination of Sr1, Sr2, Sr3, and Sr4 positions (**b**); 6-membered channels I (**c**) and II (**d**), with the channel defined by an 8-membered ring, with Sr atoms in the sitinakite structure (**e**).

The framework of Sr-exchanged sitinakite contains two independent positions of Ti1 and Ti2 and one Si1 position. The Ti1 site is associated with the Sr4 position through shared O-atoms (Figure 8a). Ti1 has five short bonds with distances ranging from 1.823–1.997 Å and one long bond of 2.120 Å. The Ti2 position is associated with Sr2 and Sr3 positions and has five short Ti-O bonds in the range of 1.819–1.992 Å and one long bond of 2.109 Å, respectively. The polyhedral volume for Ti1 and Ti2 positions is almost the same, 9.72 and 9.67 Å^3^. The Si1 site with a mean <Si-O> distance of 1.618 Å is fully populated by Si atoms only.

The extra-framework space of Sr-exchanged sitinakite is characterized by three different channels. Channel I (Figure 8c) has a hexagonal cross-section and is located along the [110] direction; channel II (Figure 8d) is also hexagonal and is located along the [100] and [010] directions; and channel III (Figure 8e) runs along the [001] direction. In Sr-exchanged sitinakite, these channels are filled by Sr^2+^ ions. There are four extra-framework sites Sr1, Sr2, Sr3, and Sr4 with occupancies of 0.17, 0.33, 0.21, and 0.28 (Figure 8b). The positions of Sr1, Sr4, Sr2, and Sr3 are located at different *z* levels parallel to the (001) plane. The anomalously short distances observed between neighboring Sr2-Sr2 and Sr4-Sr4 positions are 2.626 and 2.730 Å, respectively. Hence, the maximum theoretical occupancy of these positions is 0.5. The Sr1 position is surrounded by ten O positions, among which there are five O sites related to the framework and five extra-framework H_2_O molecules. The Sr2 position is surrounded by six framework O atoms and four H_2_O molecules. The Sr3 site is octahedrally coordinated and bound with five framework O sites and one H_2_O molecule. The Sr4 position is coordinated by five O atoms of the framework structure and four H_2_O molecules. The refined formula according to the X-ray diffraction analysis for Sr-exchanged sitinakite is Sr_1.08_[Ti_4_O_2_(O_2.16_[OH_1.84_])_4_)(SiO_4_)_2_]·4.85(H_2_O). 

**Figure 9:**
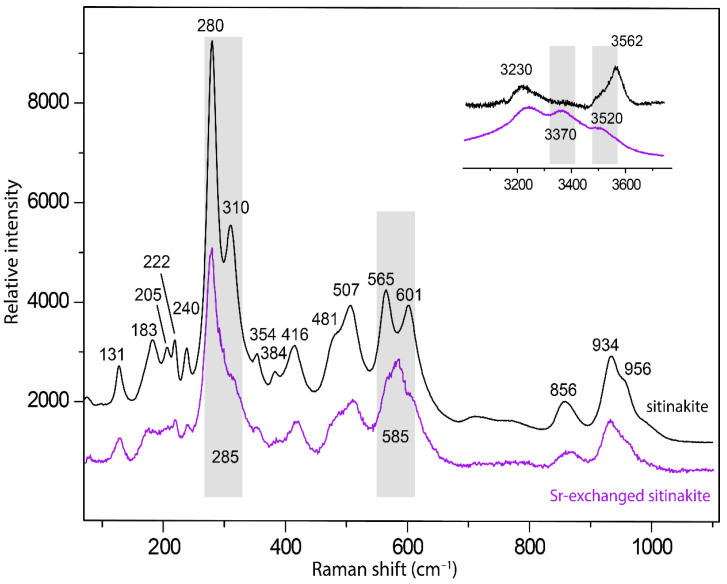
Raman spectra of the natural and Sr-exchanged sitinakite, obtained at 180 °C for 24 h. The most significant differences in position or intensity in both spectra are indicated by gray bands.

The Raman spectra of natural sitinakite and its Sr-exchanged form are shown in Figure 9. The spectrum of natural sitinakite, in general, is similar to the spectrum of sitinakite described earlier [22]. The identification of absorption bands was carried out by analogy with structurally related titanosilicates [45,46,47,48,49,50], as well as for the La-exchanged form of sitinakite [21].

The bands in the range of 850–960 cm^–1^ can be attributed to the symmetric and asymmetric stretching vibrations of Si-O bonds in the spectra of sitinakite [46]. Two bands at 565 and 601 cm^–1^ observed in the spectra of sitinakite combine into one strong band at 585 cm^–1^ in Sr-exchanged sitinakite and correspond to the asymmetric bending vibrations of Si-O bonds or the overlapping stretching vibrations of Ti-O bonds [44]. A similar band association is observed for the exchange of Na ↔ Cs и 3Na ↔ La in sitinakite [22,46]. The bands in the range of 450–510 cm^–1^ are attributed to the stretching vibrations of Ti-O bonds in TiO_6_ octahedra. The bands in the 380–450 cm^–1^ range are attributed to the symmetric bending vibrations of Si-O bonds [46,51]. The most intense bands at 281 and 310 cm^–1^ and a low-intensity band at 240 cm^–1^ correspond to the bending vibrations of Ti-O-Si and Ti-O-Ti bonds [52]. Bands in the region less than 230 cm^–1^ are related to translational modes. The bands in the region of 3240–3600 cm^–1^ correspond to the stretching vibrations of O-H bonds in H_2_O molecules and hydroxyl groups.

The incorporation of Sr^2+^ cations in the sitinakite structure induces crystal structure transformations, which affects the Raman spectra, crystal structure, and PXRD patterns.

Compared to the original sitinakite, the Raman spectrum of the Sr-exchanged sitinakite showed significant changes: the bands at 565 and 601 cm^–1^ merged into one at 585 cm^–1,^ and the intensity of the band at 310 cm^–1^ decreased significantly. Significant changes in the stretching range of H-O-H include the appearance of a new band at 3370 cm^–1^ and a shift in the 3520 cm^–1^ band to the 3562 cm^–1^ region. These changes are similar to the processes observed for La-sitinakite [22] and are associated with a change in the hydrogen bonding scheme for Sr^2+^ connected with the extra-framework cations.

**Figure 10:**
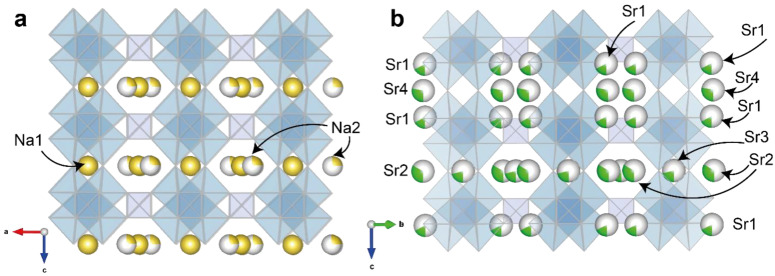
Positions of out-of-frame cations in the starting Na1 and Na2 sitinakite (**a**) and positions of Sr1, Sr2, Sr3, and Sr4 in the structure of Sr-exchanged sitinakite (**b**).

2Na^+^↔Sr^2+^ + □ substitution leads to the ordered incorporation of Sr (four Sr positions with partial occupancy instead of two Na) into the sitinakite structure (Figure 10a,b), causing a decrease in the general symmetry of *P*4_2_/*mcm* to orthorhombic *Cmmm*, as well as the appearance of additional lines in the powder diffraction pattern (see below), corresponding to a change in the unit cell parameters (*a* = 7.8158(2), *c* = 12.0248(5) Å) to (*a* =10.9784(6), *b* = 10.9781(7), *c* = 11.8861(7) Å).

**Figure 11:**
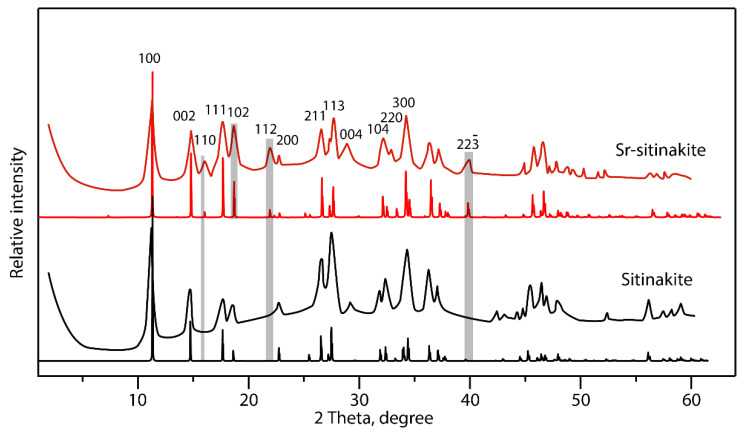
Na- and Sr-exchanged diffraction patterns of sitinakite (sample T-250) in comparison with the theoretical ones (gray lines show the main differences in the diffractograms for Na- and Sr-forms).

The diffraction patterns of the Sr-exchanged sitinakite samples show good agreement with the theoretical ones (Figure 11). The introduction of Sr^2+^ leads to the appearance of additional peaks that are absent in the original sitinakite: (110), (112), and ($22\bar{3}$) at 2θ angles of 15.70, 21.56, and 39.09°, respectively. The intensity of peak (100) at 11.09 °2θ decreases upon Sr incorporation.

Previously, the crystal structures of synthetic Sr-exchanged sitinakite were investigated using the Rietveld method on powder samples and are given in Tripathi et al. [53]. It was shown that in some cases, there is a symmetry reduction by the scheme *P*4_2_/*mcm* → *Cmmm*. Our SC XRD data using natural materials for ion exchange confirm the fact of this transition and describe in detail both the coordination environment and the occupancies of all independent channel positions of Sr.

### 3.3. Phase Transformations of Sr-Exchanged Sitinakite during Heat Treatment

**Figure 12:**
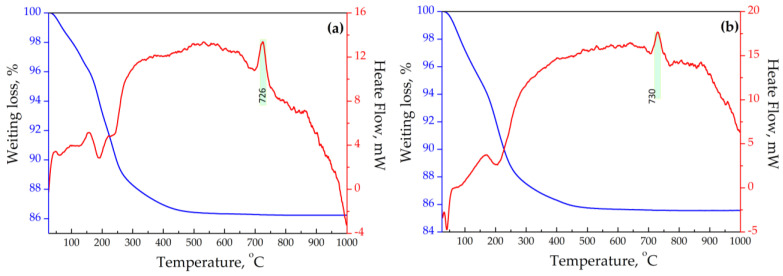
DTA and TG curves of synthesized titanosilicates: (**a**)—sample T-250-Sr and (**b**)—sample T-210-Sr.

The curves of the differential thermal analysis of the exchanged forms of sitinakite with different crystallinity are presented in Figure 12. It can be seen that the thermal behavior of Sr-exchanged sitinakite samples with different crystallinity in the temperature range of 20–1000 °C is identical. The exothermic peak with a maximum at 725 °C on the DTA curves is attributed to the polymorphic transition of anatase to rutile.

**Figure 13:**
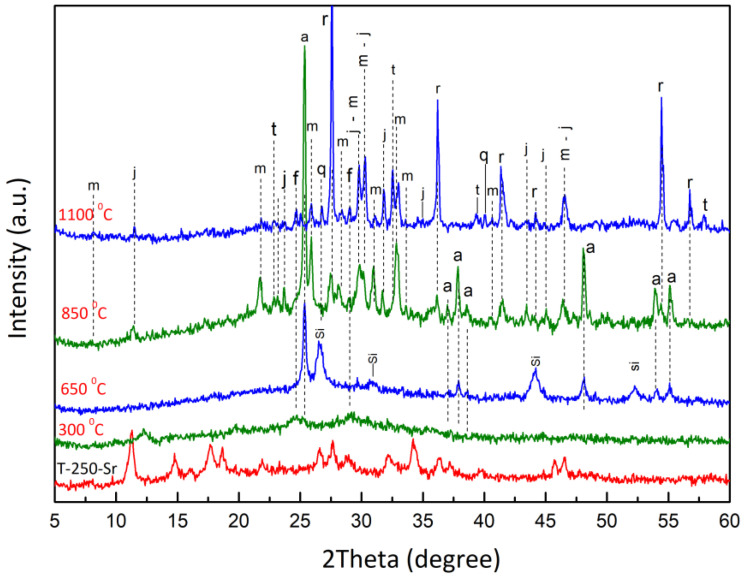
Phase transformations of Sr-exchanged sitinakite (T-250-Sr) (a—anatase; r—rutile; m—matsubaraite, j—jeppeite; t—tausonite; q—quartz; f—freidenbergite; Si—metasilicate).

Figure 13 shows the diffraction patterns of Sr-exchanged sitinakite calcined at temperatures of 300, 650, 850, and 1100 °C. The incorporation of Sr^2+^ into the lattice of sitinakite reduces its temperature stability. On the diffraction pattern of the sample calcined at 300 °C, only anatase is clearly identified. When heated to 650 °C, anatase is the main phase, with an admixture of strontium metasilicate—SrSiO_3_ (PDF Card No 00-034-0099). The exothermic peak at 725 °C on the DTA curves is attributed to matsubaraite phase crystallization. At 850 °C, the diffraction patterns reflections of rutile, matsubaraite (Sr_4_Ti_5_[Si_2_O_7_]_2_O_8_), jeppeite (SrTi_6_O_13_), and tausonite (SrTiO_3_) are fixed and the reflections of metasilicate disappear. At the temperature of 1100 °C, the crystallinity of jeppeite and tausonite phases increases, and these phases can be clearly identified. The absence of the strontium metasilicate phase is probably due to its metastability and decomposition to quartz, the reflections of which are present in the X-ray diagram.

Based on the data on the thermal analysis and phase composition of Sr^2+^ sitinakite, a variant of temperature treatment for the material to obtain ceramics is proposed, which includes sintering at 1100 °C at a rate of 10 °C/min, with exposure for 4 h at this temperature and rapid cooling in air.

### 3.4. Leaching Test

**Figure 14:**
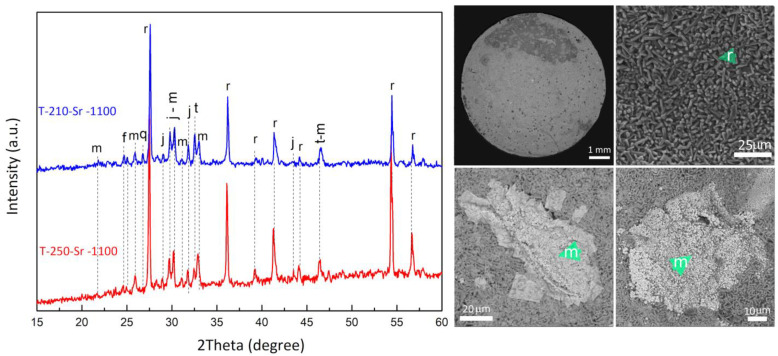
Phase composition and microstructure of ceramics obtained by calcined Sr-substituted sitinakite at 1100 °C (r—rutile; m—matsubaraite; j—jeppeite; t—tausonite; f—freidenbergite; q—quartz).

The proposed sintering scheme was favorable, which allowed the formation of dense ceramics (Figure 14). According to the SEM data, the ceramic material in the main mass is represented by rutile, in which matsubaraite aggregates are distributed. The aggregates are bound together by melt with a composition similar to matsubaraite. The phase composition of ceramic samples T-250-Sr and T-210-Sr is completely identical. The leaching results showed that treatment with distilled water for 3 days leads to insignificant desorption of Sr, which is less than 0.01% (Table 3). The calculated values of the normalized leaching rate of Sr from samples T-250 and T-210 were 2.82 × 10^–5^ and 1.05 × 10^–5^g/(m^2^·day), respectively. 

In local and international standards, leaching tests are run in aqueous media at temperatures of 25 and 90 °C for 1–28 days [54,55,56]. We conducted experiments on both the aqueous leaching of strontium ceramics and leaching in aggressive media—acids.

The desorption of Sr from ceramics during acid leaching over 3 days was much higher than in aqueous media and reached 0.17–0.19%. The leaching rate slowed down slightly on the third day (Table 3). Probably, this is connected with the leaching of Sr from phases located on the surface of the ceramics. At the same time, the strontium phases located in the volume of the ceramics were not accessible. 

Comparison with some materials showed that the obtained ceramics are characterized by low values for their normalized leaching rate (Table 4). Titanosilicate sintering results in the formation of a stable matrix for Sr fixation.

## 4. Conclusions

Na-titanosilicate with a sitinakite structure was obtained from hydrated sludge—the waste of the fluorammonium enrichment of quartz–leucoxene concentrates—by means of hydrothermal autoclave synthesis at temperatures of 210 and 250 °C. A decrease in the crystallinity of the material does not affect the sorption capacity of titanosilicate with respect to Sr cations, which is 107–112 mg/g. 

Changes in the configuration of cations in the structure channels and the hydrogen bonding scheme during the ion exchange of initial sitinakite for Sr^2+^ cations were studied through the use of PXRD and SCXRD. These changes were also confirmed through the use of Raman spectroscopy. The incorporation of Sr^2+^ into the structure of sitinakite according to the scheme 2Na^+^ → Sr^2+^ results in an ordering of four Sr positions Sr1, Sr2, Sr3, and Sr4 at different z-levels inside the channels of the structure. This mechanism is accompanied by a decrease in symmetry from *P*4_2_/*mcm* (*a* = 7.8158(2), *c* = 12.0248(5) Å) in sitinakite to *Cmmm* (*a* = 10.9784(6), *b* = 10.9781(7), *c* = 11.8861(7) Å) for its Sr-exchanged form.

Cold pressing and sintering at 1100 °C for 4 h of the Sr-exchanged form of sitinakite resulted in a ceramic for cation immobilization. It was found that the phase composition of the obtained ceramics does not depend on the crystallinity of the starting sitinakite. The main phases of the obtained ceramic material based on Sr-exchanged sitinakite are matsubaraite (matsubaraite, Sr_4_Ti_5_[S_i2_O_7_]_2_O_8_), jeppeite (SrTi_6_O_13_), tausonite (SrTiO_3_), and rutile. Leaching experiments showed the efficiency of fixation of Sr cations in the ceramic matrix; extraction into water does not exceed 0.01%, and desorption in 1 M HNO_3_ solution is only 0.19%, within 3 days. Thus, the synthesized titanosilicate is characterized by both high sorption capacity with respect to Sr and the ability to reliably fix it in the ceramic matrix. 

Reducing the synthesis temperature of sitinakite from 250 to 210 °C does not affect the sorption capacity and leaching test results of ceramics, which will lower the costs involved in its industrial production.

## Figures and Tables

**Table 1:** Crystal data and structure refinement for Sr-exchanged sitinakite.

Temperature/K	293 (2)
Crystal system	Orthorhombic
Spacegroup	*Cmmm*
*a*/Å	10.9784 (6)
*b*/Å	10.9781 (7)
*c*/Å	11.8861 (7)
α = β = γ/°	90
Volume/Å^3^	1432.54 (15)
*Z*	4
*ρ*_calc_g/cm^3^	2.985
μ/mm^−1^	6.393
F(000)	1231.0
Crystal size/mm^3^	0.14 × 0.14 × 0.14
Radiation	Mo*K*α (λ = 0.71073)
2Θ range for data collection/°	5.248 to 54.99
Index ranges	−14 ≤ h ≤ 14, −14 ≤ k ≤ 14, −14 ≤ l ≤ 15
Reflections collected	8224
Independent reflections	964 [*R*_int_ = 0.0414, *R*_sigma_ = 0.0139]
Data/restraints/parameters	964/0/95
Goodness-of-fit on F^2^	1.095
Final R indexes [I ≥ 2σ (I)]	*R*_1_ = 0.0596, w*R*_2_ = 0.1583
Final R indexes [all data]	*R*_1_ = 0.0631, w*R*_2_ = 0.1613
Largest diff. peak/hole/e Å^−3^	1.84/−1.27

**Table 2:** Chemical composition of the synthesized Na-sitinakite and its Sr-exchanged form.

Sample	Wt., % ± Δs					Sorption Capacity, mg/g	H_2_O Loss	
	SiO_2_	TiO_2_	Fe_2_O_3_	Na_2_O	SrO		at 300 °C	at 1000 °C
T-250-Na	23.1 ± 1.5	41.8 ± 1.7	2.1 ± 0.9	10.6 ± 1.5	-	-	11.3	13.3
T-250-Sr	24.4 ± 1.9	39.7 ± 1.5	3.1 ± 1.8	3.3 ± 1.1	11.7 ± 1.1	107.3	11.7	13.7
T-210-Na	23.7 ± 1.9	43.6 ± 2.0	3.1 ± 0.9	11.4 ± 1.7	-	-	13.7	15.2
T-210-Sr	22.8 ± 2.1	40.2 ± 1.7	2.5 ± 0.7	2.3 ± 0.9	12.2 ± 1.2	112.5	12.5	14.4

**Table 3:** The obtained results show reliable fixation of Sr in the ceramic matrix.

Sample	Q, mg/g	Leaching Time, day	Desorption in H_2_O, mg/g	NRi, H_2_O, g/(m^2^·day)	Desorption in HNO_3_, mg/g	NRi, HNO_3_, g/(m^2^·day)
T-250	107	1	6.9 × 10^−4^	4.9 × 10^−5^	13 × 10^−2^	10 × 10^−3^
		3	11.3 × 10^−4^	2.8 × 10^−5^	31 × 10^−2^	7.9 × 10^−3^
T-210	112	1	2.6 × 10^−4^	1.8 × 10^−5^	9 × 10^−2^	6.6 × 10^−3^
		3	4.2 × 10^−4^	1.0 × 10^−5^	20.7 × 10^−2^	4.9 × 10^−3^

**Table 4:** Normalized leaching rate of sitinakite-based ceramics compared to other materials used for Sr immobilization.

Sample	NRi g/(m^2^·day)	Conditions: Temperature (°C), Duration (days)	Reference
Sitinakite-based Sr ceramics	2.8 × 10^−5^–1.0 × 10^−5^	25, 3	Our data
Perovskite type SrTiO_3_	2 × 10^−2^–6 × 10^−2^	90, 28	[57]
Synroc D	0.1 × 10^−4^	90, 28	[58]
HIP-tailored ceramics	4 × 10^−3^	90, 28	[59]
Synroc MRS	10^−2^	90, 28	[60]
Apatite phosphate ceramics	10^−3^–10^−4^	90, 28	[61]
Apatite glass-ceramics	6.9 × 10^−4^	90, 28	[62]
Sr_0.5_Zr_2_(PO_4_)3-SmPO_4_ dual-phase ceramics	10^−4^	90, 42	[63]
Sodium zirconium phosphate	10^−3^	90, 7	[64,65]
Ceramic waste form (SrTiO_3_)	29.8 × 10^−2^	90, 7	[66]

## Data Availability

Data are contained within the article.

## Appendix A

**Table A1:** Fractional atomic coordinates (×10^4^) and equivalent isotropic displacement parameters (Å^2^ × 10^3^) for Sr-exchanged sitinakite. *U_e_*_q_ is defined as 1/3 of the trace of the orthogonalised U_IJ_ tensor.

Atom	*x*	*y*	*z*	*U_(eq)_*
Ti1	3599(2)	5000	1534.0(14)	12.6(4)
Ti2	5000	3613(2)	3470.0(14)	14.1(4)
Si1	2500	2500	2507(3)	27.5(7)
Sr2	5000	1196(4)	5000	18.5(15)
Sr4	1245(10)	5000	0	49(3)
O5	6156(9)	5000	3303(7)	12.5(17)
O4	5000	6144(9)	1710(8)	21(2)
O2	2459(9)	3721(6)	1739(7)	35.4(18)
O1	3701(6)	2493(6)	3289(6)	20.7(14)
Sr1	5000	1401(4)	1644(5)	12.9(17)
O3	3539(17)	5000	0	44(4)
O6	5000	3566(11)	5000	33(4)
O8	6190(30)	0	3387(13)	120(12)
O9	5000	0	1733(13)	155(11)
Sr3	2500	2500	5000	34(3)
O7	2928(17)	0	5000	54(4)
O10	1110(20)	2649(14)	0	155(11)

**Table A2:** Anisotropic displacement parameters (Å^2^ × 10^3^) for Sr-exchanged sitinakite. The anisotropic displacement factor exponent takes the form: −2π2[h2a*2U_11_ + 2hka*b*U_12_ + …].

Atom	*U_11_*	*U_22_*	*U_33_*	*U_23_*	*U_13_*	*U_12_*
Ti1	21.7(19)	7.3(15)	8.8(8)	0	−1.8(8)	0
Ti2	16(2)	16.5(17)	9.9(8)	1.7(7)	0	0
Si1	25(3)	5.6(19)	51.7(16)	0	0	−3.9(9)
Sr2	26(3)	5.1(18)	24(2)	0	0	0
Sr4	82(8)	38(5)	28(4)	0	0	0
O5	14(5)	10(4)	13(3)	0	5(3)	0
O4	36(7)	8(4)	20(3)	7(3)	0	0
O2	43(6)	14(3)	49(4)	−4(3)	1(4)	−11(2)
O1	23(4)	5(3)	34(3)	11(2)	−3(3)	0.6(19)
Sr1	18(4)	7(3)	14(3)	−1.7(17)	0	0
O3	59(14)	58(12)	14(5)	0	0	0
O6	78(14)	15(7)	5(4)	0	0	0
O8	310(40)	12(7)	39(9)	0	35(14)	0
O9	410(30)	45(6)	15(4)	0	0	0
Sr3	23(4)	68(7)	11(3)	0	0	−4(4)
O7	55(11)	84(12)	24(6)	0	0	0
O10	410(30)	45(6)	15(4)	0	0	0

**Table A3:** Bond lengths for Sr-exchanged sitinakite.

Atom	Atom	Length/Å	Atom	Atom	Length/Å
Ti1	Ti1 ^1^	3.076(5)	Sr2	O6	2.602(14)
Ti1	Ti2	3.1592(17)	Sr2	O8 ^8^	2.666(19)
Ti1	Ti2 ^1^	3.1592(17)	Sr2	O8 ^7^	2.666(19)
Ti1	Sr4	3.163(9)	Sr2	O8 ^6^	2.666(19)
Ti1	O5 ^1^	2.120(8)	Sr2	O8	2.666(19)
Ti1	O4 ^1^	1.997(6)	Sr2	O7	2.627(16)
Ti1	O4	1.997(6)	Sr2	O7 ^6^	2.627(16)
Ti1	O2 ^2^	1.897(7)	Sr4	Sr4 ^9^	2.73(2)
Ti1	O2	1.897(7)	Sr4	O2 ^10^	2.832(9)
Ti1	O3	1.8245(17)	Sr4	O2 ^11^	2.832(9)
Ti2	Ti2 ^1^	3.046(4)	Sr4	O2	2.832(9)
Ti2	Sr2	3.217(4)	Sr4	O2 ^2^	2.832(9)
Ti2	O5 ^1^	1.993(6)	Sr4	Sr1 ^12^	2.838(7)
Ti2	O5	1.993(6)	Sr4	Sr1 ^13^	2.838(7)
Ti2	O4 ^1^	2.109(10)	Sr4	O3	2.52(2)
Ti2	O1 ^3^	1.895(6)	Sr4	O9 ^12^	2.472(15)
Ti2	O1	1.895(6)	Sr4	O9 ^13^	2.472(15)
Ti2	Sr1	3.256(6)	Sr4	O10	2.586(15)
Ti2	O6	1.8193(17)	Sr4	O10 ^2^	2.586(15)
Ti2	Sr3 ^4^	3.5116(11)	O4	Sr1 ^1^	2.696(11)
Si1	O2 ^5^	1.622(8)	O2	Sr1 ^5^	2.706(10)
Si1	O2	1.622(8)	O1	Sr1	2.701(9)
Si1	O1	1.613(6)	O1	Sr3	2.423(7)
Si1	O1 ^5^	1.613(6)	Sr1	O8	2.89(2)
Si1	Sr1 ^5^	3.169(3)	Sr1	O8 ^8^	2.89(2)
Si1	Sr1	3.169(3)	Sr1	O9	1.542(5)
Si1	Sr3	2.963(4)	Sr1	O10 ^14^	2.527(15)
Sr2	Sr2 ^6^	2.625(8)	Sr1	O10 ^12^	2.527(15)
Sr2	O1	2.863(6)	O6	Sr3 ^4^	2.984(5)
Sr2	O1 ^4^	2.863(6)	O6	Sr3	2.984(5)
Sr2	O1 ^7^	2.863(6)	Sr3	O7 ^15^	2.784(3)
Sr2	O1 ^3^	2.863(6)	Sr3	O7	2.784(3)

^1^ 1−X,1−Y,+Z; ^2^ +X,1−Y,+Z; ^3^ 1−X,+Y,+Z; ^4^ 1−X,+Y,1−Z; ^5^ 1/2−X,1/2−Y,+Z; ^6^ 1−X,−Y,1−Z; ^7^ +X,+Y,1−Z; ^8^ 1−X,−Y,+Z; ^9^ −X,1−Y,−Z; ^10^ +X,1−Y,−Z; ^11^ +X,+Y,−Z; ^12^ 1/2−X,1/2−Y,−Z; ^13^ −1/2+X,1/2+Y,+Z; ^14^ 1/2+X,1/2−Y,+Z; ^15^ 1/2−X,1/2−Y,1−Z.

**Table A4:** Chemical composition of aggregates in the co-products of sitinakite synthesis.

Sample	Wt., % ± Δs			
	SiO_2_	TiO_2_	Fe_2_O_3_	Na_2_O
T-250-Na	11.0 ± 2.5	12.5 ± 2.1	1.0 ± 0.5	12.4 ± 1.9
T-210-Na	18.4 ± 3.5	19.5 ± 3.5	2.1 ± 0.9	10.8 ± 1.5
